# Chlorine Tolerance and Cross-Resistance to Antibiotics in Poultry-Associated *Salmonella* Isolates in China

**DOI:** 10.3389/fmicb.2021.833743

**Published:** 2022-02-04

**Authors:** Xingning Xiao, Li Bai, Sheng Wang, Lisha Liu, Xiaoyun Qu, Jianmin Zhang, Yingping Xiao, Biao Tang, Yanbin Li, Hua Yang, Wen Wang

**Affiliations:** ^1^State Key Laboratory for Managing Biotic and Chemical Threats to the Quality and Safety of Agro-Products, Ministry of Agriculture (MOA) Laboratory of Quality and Safety Risk Assessment for Agro-Products (Hangzhou), Institute of Agro-Product Safety and Nutrition, Zhejiang Academy of Agricultural Sciences, Hangzhou, China; ^2^Key Laboratory of Food Safety Risk Assessment, National Health Commission of the People’s Republic of China, China National Center for Food Safety Risk Assessment, Beijing, China; ^3^College of Food and Pharmaceutical Sciences, Ningbo University, Ningbo, China; ^4^College of Veterinary Medicine, South China Agricultural University, Guangzhou, China; ^5^Department of Biological and Agricultural Engineering, University of Arkansas, Fayetteville, AR, United States

**Keywords:** poultry, bacterial resistance, sodium hypochlorite (NaClO), *qacEΔ1*, efflux pump

## Abstract

Chlorine disinfectants have been widely used in the poultry supply chain but this exposure can also result in the development of bacterial tolerance to chlorine and this is often linked to antibiotic cross-resistance. The objectives of this study were to investigate sodium hypochlorite (NaClO) tolerance of *Salmonella* isolated from poultry supply chains and evaluate cross-resistance. We collected 172 *Salmonella* isolates from poultry farms, slaughter houses and retail markets in China during 2019–2020. We found that *S.* Enteritidis, *S.* Kentucky, and *S.* Typhimurium constituted > 80% of our *Salmonella* isolates. Overall, 68% of *Salmonella* isolates were resistant to > 3 antibiotics and *S.* Kentucky displayed a significantly (*p* > 0.05) higher frequency (93.2%) of multidrug resistance than the other serovars. Tolerance to chlorine at MIC > 256 mg/L was detected in 93.6% of isolates (161/172) and tolerant isolates displayed higher decimal reduction times (*D* value) and less ultrastructural damage than did the suspectable strains under chlorine stress. Spearman analysis indicated significant positive correlations between chlorine tolerance (evaluated by the OD method) and antibiotic resistance (*p* < 0.05) to ceftiofur, tetracycline, ciprofloxacin and florfenicol and this was most likely due to efflux pump over-expression. The most frequently detected chlorine resistance gene was *qacE*Δ*1* (83.1%, *n* = 143) and we found a positive correlation between its presence and MIC levels (*r* = 0.66, *p* < 0.0001). Besides, we found weak correlations between chlorine-tolerance and antibiotic resistance genes. Our study indicated that chlorine disinfectants most likely played an important role in the emergence of chlorine tolerance and spread of antibiotic resistance and therefore does not completely control the risk of food-borne disease. The issue of disinfectant resistance should be examined in more detail at the level of the poultry production chain.

## Introduction

*Salmonella* is a frequent cause of gastroenteritis in humans and is an important public health concern worldwide and in China causes 9.87 M gastroenteritis cases annually (Xiao et al., 2019; Yang et al., 2020a). More than 2600 *Salmonella enterica* serovars have been identified and *S.* Enteritidis, *S.* Typhimurium, *S.* Kentucky, and *S.* Indiana are the most frequent causes of human salmonellosis in China (Andoh et al., 2016; Elnekave et al., 2018; Zeng et al., 2021). Poultry products are the most common vehicles for *Salmonella* transmission and a recent study in China found that 37.5% of poultry samples were contaminated with *Salmonella* (Yang et al., 2020b). Contamination sources include the animals when introduced into the poultry house environment and direct or cross-contamination between poultry carcasses. All these events can result in foodborne salmonellosis when improperly cooked or handled products are consumed (Xiao et al., 2019).

Chlorine disinfection is one of the most common disinfection technologies for controlling the risks of microorganisms in industrial processing (Jia et al., 2020). In this process, bacteria can survive and even reproduce in residual chlorine and are designated chlorine tolerant (Langsrud et al., 2003; Luo et al., 2020). In particular, *Salmonell*a possesses a high level of regeneration capacity in reclaimed water after chlorine disinfection (Li et al., 2013). Recent studies have focused on chlorine tolerance of bacteria in water while few studies addressed contamination on the processed poultry products (Roy and Ghosh, 2017; Bhojani et al., 2018; Wang et al., 2019; Luo et al., 2020). In China, 50–100 mg/L of sodium hypochlorite (NaClO) is commonly used in poultry processing and environmental disinfection while the inactivation of *Salmonella* was limited (< 1 Log) and could be due to chlorine tolerance (Jun et al., 2013; Lee et al., 2014; Xiao et al., 2019).

Current studies have also demonstrated that long-term antibiotic usage during animal breeding has led to a marked increase in the levels of antibiotic resistance (Zeng et al., 2021). Antibiotic susceptibility testing of the 318 poultry-associated *Salmonella* revealed that only 5 (1.6%) were susceptible to all 22 tested antibiotics, while 191 (60.1%) exhibited multidrug resistance. Poultry frequently harbor antibiotic-resistant *Salmonella* isolates that can be transmitted to humans via the food chain (Yang et al., 2020a). The emergence of quinolone, tetracycline and extended-spectrum β-lactam-resistant *Salmonella* is a serious public health concern (Mechesso et al., 2020). Cross-resistance between disinfectants and antibiotics is also becoming widespread for bacterial pathogens (Carey and McNamara, 2015; Puangseree et al., 2021). For example, a higher level of chlorine tolerance in antibiotic-resistant *Escherichia coli* (*E. coli*) was found when compared to antibiotic-susceptible strains (Templeton et al., 2013). Bacterial exposure to chlorine increases the expression of efflux pumps and activates *qac* genes that also export chloramphenicols, sulfonamide and β-lactams (Karumathil et al., 2014). However, chlorine tolerance and cross-resistance of *Salmonella* that are present in the poultry supply chain have not been fully investigated.

The focus of the current study was to investigate (i) chlorine tolerance and antibiotic resistance in *Salmonella* spp. isolated from poultry supply chains and (ii) to determine the level of cross-resistance in these isolates. This study examined on a broad scale the important role played by chlorine disinfectants as a non-antibiotic selective pressure for the emergence and spread of antibiotic resistance.

## Materials and Methods

### *Salmonella* Isolates and Serovar Identification

We isolated 172 *Salmonella* spp. from poultry supply chains as follows: poultry farms (*n* = 10), slaughter houses (*n* = 102) and retail markets (*n* = 60) in Guangzhou, Shandong and Zhejiang provinces during 2019–2020. *Salmonella* isolates were subcultured and serotyped by slide agglutination using commercial O and H antisera to distinguish *Salmonella* serovars (SSI-Diagnostica, Copenhagen, Denmark; Tianrun Bio-Pharmaceutical, Ningbo, China) in accordance with the White-Kauffmann-Le Minor scheme (Grimont and Weill, 2007; Slotved et al., 2016). All isolates were stored in brain heart infusion (BHI) broth (Becton Dickinson, Franklin Lakes, NJ, United States) containing 20% glycerol at −80°C until use.

### NaClO Tolerance Determinations

#### Determination of Minimum Inhibitory Concentrations and Optical Density

The MIC values for NaClO in the *Salmonella* isolates were measured using broth microdilution. The *E. coli* ATCC 29522 and *S.* Enteritidis CVCC 1806 were included as controls for susceptibility testing. Bacterial suspensions were prepared by suspending 3–5 individual overnight colonies from trypticase soy (TSA) agar (Becton Dickinson) plates into 3 mL of 0.9% saline, equivalent to the turbidity of a 0.5 McFarland standard. The 0.5 McFarland inoculum suspensions were further diluted at 1:100 in Mueller-Hinton (MH) broth (Becton Dickinson). NaClO stock solutions containing 56.8 mg/mL chlorine was purchased from Sangon Biotech (Shanghai, China) and were prepared by dilution in sterile Milli-Q water (PALL, Buckinghamshire, United Kingdom). Chlorine concentrations were determined using a Palintest ChlorSense meter (Gateshead, United Kingdom). Preliminary tests indicated the MIC of *S.* Enteritidis CVCC 1806 to NaClO was 256 mg/L and tolerance to NaClO was defined as > 256 mg/L. These MIC assays for chlorine tolerance were performed in 96-well microtiter test plates containing 100 μL NaClO solution and were inoculated with 100 μL of suspended bacterial cultures to a final inoculum density of 5 Log CFU/mL per well. The plates were sealed using a perforated plate seal and incubated at 37°C for 24 h. The MIC values of NaClO were recorded as the lowest concentration of NaClO where no visible growth was observed. Control wells containing bacteria and lacking chlorine and wells with MH broth were used as positive and negative controls, respectively. Each assay was repeated three times on different days. Cell growth in the plates was also quantified by measuring the optical density at 600 nm (OD_600_) (Bernardez and de Andrade Lima, 2015; Yang et al., 2020a) using a Polarstar spectrophotometer (Omega, Ortenberg, Germany).

#### Inactivation Kinetics

Chlorine susceptible and tolerant isolates were selected based on the MIC and OD_600_ tests and then separately incubated in BHI at 37°C for 24 h and cultured to approximately 9 Log CFU/mL. The initial inoculum level was 5 ± 0.2 Log CFU/mL. Cell suspensions were treated with 100 mg/L of NaClO for 0, 30, 60, 90, and 120 min. Then the suspension was put into a sterile centrifuge tube containing sodium thiosulfate (Na_2_S_2_O_3_) to instantaneously quench the residual disinfectant and 10-fold diluted in buffered peptone water (Becton Dickenson) and a 50 μL portion of appropriate dilutions were plated in duplicate onto TSA agar plates using a spiral plater (WASP 2, Don Whitley Scientific, Shipley, United Kingdom). The plates were incubated at 37°C for 18 h. Colonies on TSA agar plates were enumerated by a ProtoCOL 3 automated colony counter (Synbiosis, Cambridge, United Kingdom). The limit of detection was one colony per 50 μL sample (1.3 Log CFU/mL). Each treatment was repeated three times on different days and duplicate plates were used in microbial tests for each sample.

The decimal reduction time (*D* value) was used as an index to evaluate the bacterial inactivation rate vs. NaClO treatment as previously described (Luo et al., 2020). *D* values were calculated at the initial linear portion of survivor plots assuming the logarithmic numbers of bacterial growth were a linear function of the isothermal treatment time (Ahn et al., 2007). The log-linear model was used to investigate the pattern of pathogen inactivation after NaClO treatment as follows:


$$\log N_{t}=\log N_{0}-\frac{t}{D}$$

where *N*_*t*_ (CFU/mL) is the bacterial population at time *t* (s), *N*_0_ (CFU/mL) is the initial bacterial population, and *D* is the decimal reduction time (min) at a specific treatment condition.

#### Membrane Damage

Bacterial viability and cell properties between NaClO tolerant and susceptible bacteria were measured using flow cytometry (FCM) to quantify viable, dead and injured cells after treatment. *Salmonella* cell suspensions (8 Log CFU/mL) were treated with NaClO at 100 mg/L for 0, 10, and 30 min. A commercial live/dead staining kit (BacLight, L7012, Molecular Probes, Carlsbad, CA, United States) was used to distinguish living, dead, and damaged states of bacteria. In brief, 1 mL of bacteria solution was mixed with 1.5 μL SYTO 9 and 1.5 μL propidium iodide (PI) dyes. The mixture was kept in darkness at room temperature for 15 min. FCM tests were performed using a Accuri C6 flow cytometer (Becton Dickinson) equipped with lasers emitting at 488 and 640 nm. Some influences of impurities (such as large particles) on bacterial detection were excluded according to the difference in forward and side light scatter and 10,000 events were collected. The non-treated stained and non-stained cells served as control samples to adjust the detectors (Barros et al., 2021).

Cell structure damage under NaClO stress was observed using scanning electron (SEM, Hitachi 8010, Ibaraki, Japan) and transmission electron (TEM, Hitachi 7650, Ibaraki, Japan) microscopy. *Salmonella* cell suspensions (8 Log CFU/mL) were harvested by centrifugation following NaClO treatments (see below) and fixed with a 2.5% glutaraldehyde (Sangon Biotech, Shanghai, China) solution overnight at 4°C. The cells were centrifuged and the pellets were washed three times with 0.1 M sodium phosphate buffer solution. Each resuspension was serially dehydrated with 25, 50, 75, 90, and 100% ethanol, respectively, and examined using SEM and TEM as previously described (Tyagi and Malik, 2012).

### Antibiotic Resistance Determination

Bacterial suspensions were prepared by suspending 3–5 individual colonies grown at 37°C for 18 h on TSA into 3 mL 0.9% saline, equivalent to the turbidity of a 0.5 McFarland standard. The 0.5 McFarland inoculum suspensions were further diluted at 1:100 in MH. A panel of antibiotic agents was reconstituted by adding 200 μL/well of the inoculum and incubated at 37°C for 18 h. The *E. coli* ATCC 25922 was used as the reference strain. Antibiotic susceptibility testing was performed using the broth microdilution method with the commercial Gram-negative antibiotic panel (Biofosun, Fosun Diagnostics, Shanghai, China) consisting of ampicillin (resistant, AMP ≥ 32 μg/mL), amoxicillin/clavulanate (resistant, AMC ≥ 32 μg/mL), cefotaxime (resistant, CTX ≥ 4 μg/mL), meropenem (resistant, MEM ≥ 4 μg/mL), amikacin (resistant, AMK ≥ 64 μg/mL), gentamicin (resistant, GEN ≥ 16 μg/mL), colistin (resistant, CS ≥ 2 μg/mL), ceftiofur (resistant, CEF ≥ 8 μg/mL), ciprofloxacin (resistant, CIP ≥ 1 μg/mL), sulfamethoxazole (resistant, T/S ≥ 4/76 μg/mL), tetracycline (resistant, TET ≥ 16 μg/mL), tigecycline (resistant, TIG ≥ 8 μg/mL), and florfenicol (resistant, FFC ≥ 16 μg/mL). The breakpoints for each antibiotic agent were set by the 2019 Clinical and Laboratory Standards Institute (CLSI) and the 2017 European Committee on Antimicrobial Susceptibility Testing (EUCAST). Bacteria that were susceptible to all 13 antibiotics were classified as drug-suspectable and bacteria resistant to 1 or 2 classes of antibiotics were termed drug resistant. Multidrug resistance was defined as resistance to 3 or more different classes of antibiotics.

### Chlorine and Antibiotic Resistance Genes in *Salmonella* Isolates

#### DNA Extraction

DNA was extracted from pure *Salmonella* colonies using a QIAamp1 Mini DNA Extraction Kit (Qiagen, Hilden, Germany) according to the manufacturer’s instructions.

#### PCR Detection of Chlorine and Antibiotic Resistance Genes

Antibiotic resistance and chorine resistance genes were selected based on previous studies (Hu et al., 2018, 2021; Chen et al., 2021) and the use of the ResFinder database.^1^ We screened for the presence of -eight chlorine resistance genes (*exbD, motB, qacE, qacEΔ1, qacF, sugE, ybhR*, and *yedQ*) and ten antibiotic resistance genes (*bla*_*OXA*,_ *bla_*CTX*_, bla_*TEM*_, qnr_*B*6_, qnr_*S*7_, tetG, tetA, floR, cat86*, and *cmx*) using PCR assays (Table 1) as previously described (Wu et al., 2015). Amplicons were electrophoresed in 1% gel agarose gels containing EtBr and were visualized under UV light.

**TABLE 1:** Frequency of chlorine tolerance and antibiotic resistance genes determined by PCR among *Salmonella* isolates (*n* = 172).

	Gene	Primer sequence (5′→3′)	Amplicon size (bp)	No. (%)
Chlorine resistance	*exbD*	CGTTAGCGACGGTAGATGTGA	230	43.6
		CAACGTCTCGTAATCGACGGTT		
	*motB*	GGTGATCTCGACCAGTTGATAG	363	29.7
		GCCGACGACACGTAACACTT		
	*qacE*	GGGCGTAGTATGGTTACTTGTTG	196	0.006
		TCCAAGGCCTGACCACATGA		
	*qacE*Δ*1*	GTTGGCGAAGTAATCGCAACATC	200	83.1
		AGCAACCAGGCAATGGCTGTA		
	*qacF*	GCGATTGCTTGTGAAGTTATTGCAAC	175	59.9
		TACCCGCACCCGACCAAATG		
	*sugE*	AAGTACACCGACGGCTTCACT	184	4.1
		TGGATTCGCCGAACAGGATGA		
	*ybhR*	GCCAACTACCTGCAACAGATC	277	20.3
		GTACGGCTTTGCCGATGAAGAT		
	*yedQ*	CGCACAACTCCTCATCAATGG	228	0
		CAGTGACCTGTGGCTCTGT		
Antibiotic resistance	*bla*_*OXA*_	GAAGCACACACTACGGGTGTT	342	0
		GGTACCGATATCTGCATTGCCATA		
	*bla*_*CTX*_	GCACGTCAATGGGACGATGT	326	65.1
		TCGCTGCACCGGTGGTAT		
	*bla*_*TEM*_	CGGATGGCATGACAGTAAGAG	324	76.7
		GCAGAAGTGGTCCTGCAACT		
	*qnr*_*B6*_	CAGTGCGCTGGGCATTGAA	302	8.7
		CCGAATTGGTCAGATCGCAATG		
	*qnr*_*S7*_	CAGCGACTTTCGACGTGCTA	305	37.8
		CCCTCTCCATATTGGCATAGG		
	*tetG*	GCTAGAGCTGTTGAACGAGGTT	181	1.7
		CTCATTCTCTTCGGGTAGCGA		
	*tetA*	TGTGCTCGGTGGGCTGAT	165	73.3
		AACGAAGCGAGCGGGTTGA		
	*floR*	GTCCGCTCTCAGACAGAATC	246	61.0
		GCACGAACGCCAGAATCGA		
	*cat86*	GCTGTGTAGCCGATATTGAAAC	222	0
		GTACTTGTATGGCAACGGGCAAA		
	*cmx*	CATCCTGCTCGCCGTACT	232	0
		CACTCTCCTGTCCATGAGGAT		

### Statistical Analysis

Cross-resistance between chlorine and antibiotics was assessed by comparing antibiotic MIC data with OD_600_ values growth in the presence of NaClO (128 mg/L) using the non-parametric Spearman correlation test in SPSS Statistics 20 software (IBM, Chicago, IL, United States). The Spearman coefficients ranged from –1 to + 1 indicating no association (*r* = 0) to monotonic relationships (*r* = –1 or + 1) (Schober et al., 2018). Significant differences were set at *p* < 0.05.

## Results

### Diversity of *Salmonella* Serotypes

We identified numerous *Salmonella* serovars from the wide range of samples we collected. These included samples from 10 farms (1 serotype), 102 slaughter houses (8 serotypes) and 60 retail markets (7 serotypes). A total of 10 serotypes were identified in 172 isolates and included *S.* Enteritidis, *S.* Kentucky, *S.* Typhimurium, *S.* Indiana, *S.* Agona, *S.* Stanley, *S.* Thompson, *S.* Derby, *S.* Mbandaka, and *S.* Montevideo. The most frequent serovars in the poultry supply chain were *S.* Enteritidis (77/172, 44.8%), *S.* Kentucky (44/172, 25.6%) and *S.* Typhimurium (18/172, 10.5%) and these accounted for > 80% of the total serovars isolated (Table 2).

**TABLE 2:** Bacterial of *Salmonella* spp. isolated from poultry supply chains.

Sampling location	Number of isolates	Serotype distribution
**Farm**		
Anal swabs	10	10 Typhimurium
**Slaughter house**		
Poultry carcasses	100	43 Enteritidis, 33 Kentucky, 6 Typhimurium, 6 Indiana, 2 Thompson, 1 Derby, 1 Montevideo, 1 Stanley, 7 Unidentified
Water	2	1 Enteritidis, 1 Kentucky
**Retail market**		
Poultry carcasses	60	33 Enteritidis, 10 Kentucky, 3 Indiana, 2 Agona, 2 Typhimurium, 1 Mbandaka, 1 Stanley, 8 Unidentified
Total	172	77 Enteritidis, 44 Kentucky, 18 Typhimurium, 9 Indiana, 2 Agona, 2 Stanley, 2 Thompson, 1 Derby, 1 Mbandaka, 1 Montevideo, 15 Unidentified

### NaClO Tolerance Evaluation

#### Minimum Inhibitory Concentrations and OD_600_ Determination

**FIGURE 1:**
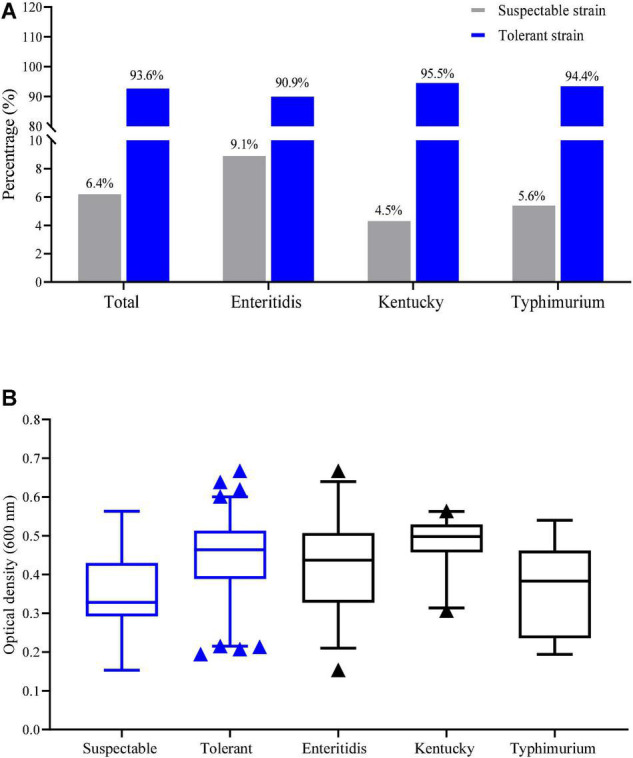
Distribution of tolerance isolates by NaClO based on **(A)** MIC and **(B)** OD_600_ values.

Tolerance to NaClO at MIC > 256 mg/L was detected in 93.6% of our *Salmonella* isolates (161/172). Tolerance based on the MIC values in the serovars *S.* Enteritidis, *S.* Kentucky and *S.* Typhimurium isolates were 90.9, 95.5, and 94.4%, respectively (Figure 1A). The average of OD_600_ values for tolerance (MIC > 256 mg/L) and susceptibility were 0.44 ± 0.10 and 0.33 ± 0.09, respectively and this difference was significant (*p* < 0.05). The average of OD_600_ values for growth in the presence of chlorine for *S.* Enteritidis, *S.* Kentucky and *S.* Typhimurium isolates were 0.42 ± 0.11, 0.48 ± 0.06, and 0.37 ± 0.11, respectively (Figure 1B). These data indicated that *S*. Kentucky displayed the highest chlorine tolerance compared with other two serotypes (*p* < 0.05).

#### Inactivation Kinetics

**FIGURE 2:**
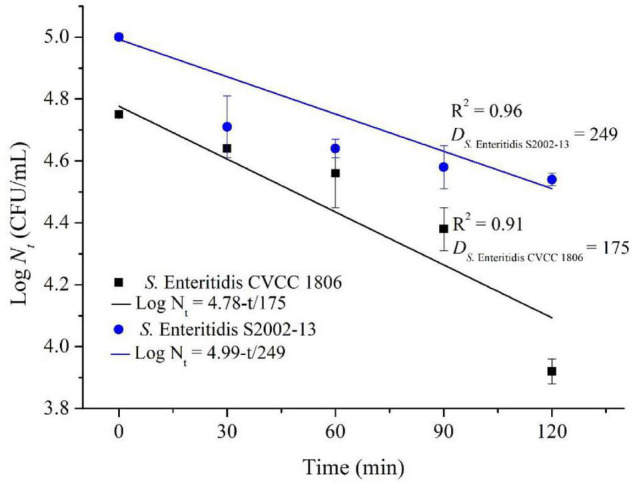
Inactivation kinetics of *S.* Enteritidis CVCC 1806 and *S.* Enteritidis S2002-13 exposed to concentration of NaClO at 100 mg/L for 0, 30, 60, 90, and 120 min.

We more closely examined the mechanisms for choline tolerance using two defined laboratory strains that were susceptible (*S.* Enteritidis CVCC 1806) and tolerant (*S.* Enteritidis S2002-13). We found that growth inhibition for these strains significantly (*p* < 0.05) differed when cultured for 120 min in the presence of NaClO resulting in 0.83 ± 0.04 (*S.* Enteritidis CVCC 1806) and 0.46 ± 0.02 (*S.* Enteritidis S2002-13) Log CFU/mL, respectively. The data were fitted to the log-linear model and the *D* values of NaClO required to inactivate 1 Log unit for *S.* Enteritidis CVCC 1806 and *S.* Enteritidis S2002-13 were 175, and 249 min, respectively (Figure 2). These data indicated that *S.* Enteritidis S2002-13 displayed reduced susceptibility to NaClO.

#### Membrane Damage

**FIGURE 3:**
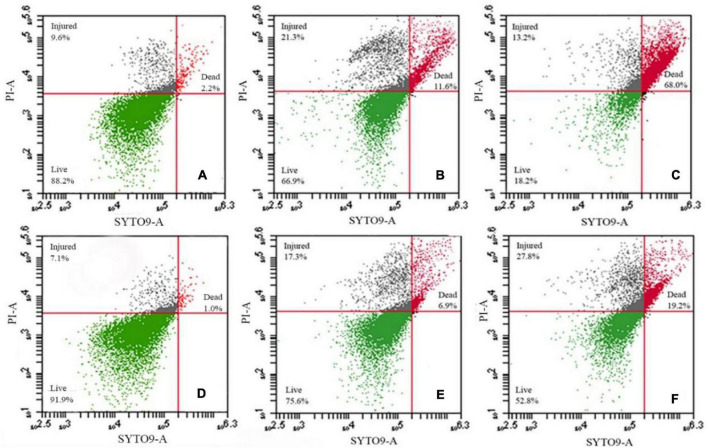
*S.* Enteritidis CVCC 1806 **(A–C)** and *S.* Enteritidis S2002-13 **(D–F)** stained with SYTO9 plus PI after treatment with 100 mg/L NaClO for 0, 10 and 30 min.

We further assessed membrane damage to the bacteria using the membrane-impermeable green fluorescent nucleic acid dye SYTO9 and chlorine-treated cells had higher levels of staining (Figures 3A,D). Exposure to NaClO for 10 min resulted in staining of 11.6% of the susceptible *S.* Enteritidis CVCC 1806 and 6.9% of tolerant *S.* Enteritidis S2002-13 cells (Figures 3B,E). Exposure for 30 min resulted in 68% of *S.* Enteritidis CVCC 1806 as positive for PI staining compared with 19.2% of *S.* Enteritidis S2002-13 (Figures 3C,F). These data clearly showed that *S.* Enteritidis S2002-13 was more tolerant to NaClO.

**FIGURE 4:**
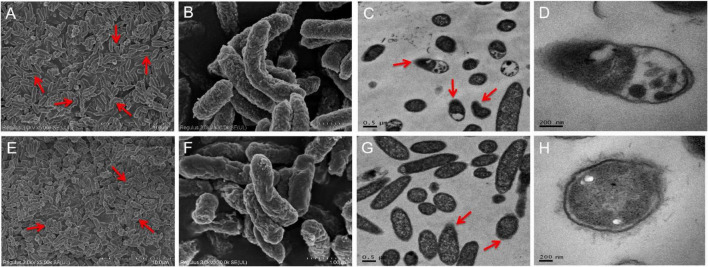
SEM and TEM photomicrographs of *S.* Enteritidis CVCC 1806 **(A–D)** and *S.* Enteritidis S2002-13 **(E–H)** after treatment with 100 mg/L NaClO for 30 min. The red arrows indicate regions of bacterial cell damage.

Ultrastructural analyses indicated the presence of severe cell ruptures for *S.* Enteritidis CVCC 1806 following NaClO treatment and cytoplasmic contents were displaced (Figures 4A–D). In contrast, structural damage was less common in *S.* Enteritidis S2002-13 and most cells showed no internal rearrangements of internal cellular content (Figures 4E–H). These data clearly demonstrated showed that *S.* Enteritidis S2002-13 was more tolerant to NaClO than *S.* Enteritidis CVCC 1806.

### Antibiotic Resistance Phenotypes

We also assessed our population for antibiotic resistance and 155 of our *Salmonella* isolates (90.1%) were resistant to at least one and 117 (68%) isolates were resistant to at least 3 antibiotics. These results were similar to a previous study of *Salmonella* isolates from poultry in China (Tang et al., 2020). A majority of the *Salmonella* isolates were resistant to AMC (71.5%), AMP (70.9%), and TET (71.5%) and the least prevalent resistance phenotype was found for MEM (0.58%). Additionally, we found no evidence for differences in antibiotic resistance with sampling location. However, resistance did vary by serovar and *S.* Kentucky displayed the highest prevalence of multidrug resistance (93.2%) than the other two most common serotypes (Table 3).

**TABLE 3:** Frequency (% of total isolates) of resistance to antibiotic agents among *Salmonella* isolates from poultry supply chains.

Antibiotic	% (No. of resistant isolates)							
	Sampling location			Predominant serotype				Total (%) (*n* = 172)
		
	Farm (*n* = 10)	Slaughter house (*n* = 102)	Retail (*n* = 60)	Enteritidis (*n* = 77)		Kentucky (*n* = 44)	Typhimurium (*n* = 18)	
**β -lactams**								
AMP	6 (60.0)	78 (76.5)	38 (63.3)		53 (68.8)	38 (86.4)	9 (50.0)	122 (70.9)
AMC	7 (70.0)	79 (77.5)	37 (61.7)		54 (70.1)	37 (84.1)	11 (61.1)	123 (71.5)
CTX	3 (30.0)	38 (37.3)	17 (28.3)		5 (6.5)	34 (77.3)	3 (16.7)	58 (33.7)
MEM	0 (0)	0 (0)	1 (1.7)		0 (0)	0 (0)	0 (0)	1 (0.58)
CEF	2 (20.0)	45 (44.1)	19 (31.7)		11 (14.3)	35 (79.5)	3 (16.7)	66 (38.4)
**Aminoglycosides**								
AMK	0 (0)	29 (28.4)	8 (13.3)		3 (3.9)	28 (63.6)	0 (0)	37 (21.5)
GEN	3 (30.0)	38 (37.3)	18 (30.0)		5 (6.5)	36 (81.8)	3 (16.7)	59 (34.3)
**Sulfonamides**								
T/S	6 (60.0)	48 (47.1)	19 (31.7)		12 (15.6)	36 (81.8)	6 (33.3)	73 (42.4)
**Quinolones**								
CIP	5 (50.0)	46 (45.1)	20 (33.3)		5 (6.5)	40 (90.9)	6 (33.3)	71 (41.3)
**Tetracyclines**								
TET	7 (70.0)	58 (56.9)	29 (48.3)		16 (20.8)	44 (100)	12 (66.7)	94 (54.7)
TIG	1 (10.0)	13 (12.7)	6 (10.0)		1 (1.3)	13 (29.6)	2 (11.1)	20 (11.6)
**Polymyxins**								
CS	4 (40.0)	12 (11.8)	18 (30.0)		25 (32.5)	2 (4.5)	4 (22.2)	34 (19.8)
**Chloramphenicol**								
FFC	6 (60.0)	47 (46.1)	22 (36.7)		6 (7.8)	38 (86.4)	8 (44.4)	75 (43.6)
DS*	1 (10.0)	10 (9.8)	6 (10.0)		11 (14.3)	0 (0)	2 (11.1)	17 (9.9)
DR**	1 (10.0)	20 (19.6)	17 (28.3)		29 (37.7)	3 (6.8)	4 (22.2)	38 (22.1)
MDR***	8 (80.0)	72 (70.6)	37 (61.7)		37 (48.1)	41 (93.2)	12 (66.7)	117 (68.0)

*DS, bacterial susceptible to all 13 antibiotics. **DR, bacteria resistant to 1 or 2 classes of antibiotic**s**. ***MDR, multidrug resistance was defined as resistance to three or more different classes of antibiotics. AMP, Ampicillin; AMC, Amoxicillin/clavulanate; CTX, Cefotaxime; MEM, Meropenem; AMK, Amikacin; GEN, Gentamicin; CS, Colistin; CEF, Ceftiofur; CIP, Ciprofloxacin; T/S, Sulfamethoxazole; TET, Tetracycline; TIG, Tigecycline; and FFC, Florfenicol.

### Determination of Chlorine and Antibiotic Resistance Genes

Our *Salmonella* isolates were also screened for the presence of genes enabling chlorine resistance and *qacE*Δ*1* gene was the most prevalent (83.1%; *n* = 143) and *qacF* (59.9%; *n* = 103), *exbD* (43.6%; *n* = 75), and *motB* (29.7%; *n* = 51) were also highly represented. We found a positive correlation between the presence of chlorine resistance genes and higher MIC for chlorine that were associated with possession of *qacEΔ1* (*r* = 0.66, *p* < 0.0001) and to lesser extents *qacF* (*r* = 0.36, *p* < 0.0001), *exbD* (*r* = 0.26, *p* < 0.0001), and *motB* (*r* = 0.19, *p* < 0.01). We also found that the antibiotic resistance genes *bla*_*TEM*_ (76.7%, *n* = 132), *tetA* (73.3%, *n* = 126), *bla*_*CTX*_ (65.1%, *n* = 112), and *floR* (61%, *n* = 105) were highly represented in our isolates from the different nodes of the poultry supply chain (Table 1).

### Association Between Chlorine and Antibiotic Resistance

**FIGURE 5:**
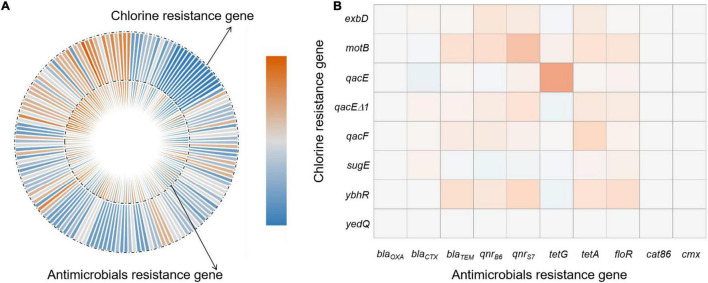
Correlations of bacterial NaClO and antibiotic l resistance genes based on **(A)** correlation coefficient and **(B)** frequency of resistance gene detection.

We compared our *Salmonella* isolates based on the antibiotic MIC values against OD_600_ values for NaClO (128 mg/L) using non-parametric Spearman correlation tests to determine whether chlorine and antibiotic resistance were correlated. We found significant positive relationships between chlorine and ceftiofur, tetracycline, ciprofloxacin and florfenicol resistance and was most likely related to the over-expression of drug efflux pumps (Table 4). *Salmonella* isolates with resistance to chlorine and antibiotics were analyzed for their content of resistance genes. In our group of 172 isolates, chlorine tolerant strains were more likely to also be antibiotic resistant (*p* < 0.05) (Figure 5A). This was especially for the *qac* genes that displayed a relatedness with correlation coefficients in the range of 0.10–0.44 (Figure 5B).

**TABLE 4:** Spearman correlation analysis for OD_600_ (NaClO) and MIC (antibiotic) measurements.

Antibiotic	β -lactams (CEF)	Quinolones (CIP)	Tetracyclines (TET)	Chloramphenicol (FFC)
Spearman correlation	0.18	0.15	0.21	0.25
*p*-value	0.01	0.03	0.03	0.001

*CEF, Ceftiofur; CIP, Ciprofloxacin; TET, Tetracycline; and FFC, Florfenicol. Significant differences, p < 0.05.*

## Discussion

### Chlorine Tolerance

There is no widely accepted scientific definition of chlorine tolerance in bacteria or for quantification of the chlorine tolerance phenotype. Previous studies have utilized MIC, inhibition zone, logarithmic removal rate and membrane damage methods for determining bacterial chlorine tolerance (Jia et al., 2020; Luo et al., 2020). The MIC method is suitable for comparison of chlorine tolerance among isolates, but it cannot reflect the inactivation effect of typical MIC value (Garcia and Pelaz, 2008). Growth inhibition can be measured using 96 well plates and chlorine potency can be judged from growth inhibition quantified using absorbance at 600 nm (Kolarević et al., 2016). In our study, we combined MIC and OD_600_ methods to evaluate chlorine tolerance. Therefore, the disadvantage that for the MIC method that it does not reflect inactivation effects at the same MIC values was overcome.

The inhibition zone method is suitable for comparison of chlorine tolerance of a large number of isolates but becomes problematic with bacteria with differing growth rates and test results are often inaccurate (Khan et al., 2016; Luo et al., 2020). A previous study had isolated 87 bacterial strains in 22 genera from drinking water and chlorine tolerance could be differentiated at the species and genus levels (Khan et al., 2016). Logarithmic inactivation rates are suitable for comparison of chlorine tolerance between studies but disinfection concentration and time have not been standardized between studies (Zeng et al., 2020). Additionally, the antimicrobial activity of disinfectants is routinely tested by determination of survival curves and this procedure is labor-intensive and time consuming (Puangseree et al., 2021).

Culture-independent flow cytometry combined with fluorescent dyes has been used to investigate the effects of chlorine disinfection on bacterial physiological properties including membrane integrity and potential and respiratory activity (Song et al., 2019). Chlorine inactivates microorganisms by reacting and damaging cellular components including cell walls, membranes and nucleic acids. Chlorine-susceptible and chlorine-tolerant bacteria possess differences in the relative percentages of fatty acids in cell walls and membranes. Chlorine tolerance has been explained by replacement of 95.7% of membranes lipids as saturated long-chain fatty acids that act to limit chlorine diffusion (Chen et al., 2012).

### Mechanisms of Chlorine Tolerance in Bacteria

Chlorine disinfection induces the expression of many functional gene families including responses to oxidative stress, DNA repair, energy metabolism, membrane damage and efflux pumps (Hou et al., 2019; Luo et al., 2020). The SOS response (a conserved response to DNA damage) triggered by oxidative stress renders bacteria chlorine resistant (Tong et al., 2021). Nutrient limitations result in quiescence of growth and metabolism that may subsequently lead to bacteria in the viable but non-culturable (VBNC) state for chlorine stress adaptations (Zhu et al., 2022). *E. coli, Enterococcus and Salmonella* can enter into a VBNC state after disinfection, and *Salmonella* was more resistant to chlorine in reclaimed water (Li et al., 2013). In addition, membrane permeability is a primary barrier to uptake of foreign or extracellular DNA and NaClO exposure alters the expression of cell membrane-related genes and especially those regulating cell membrane permeability as has been demonstrated in *Pseudomonas* spp. (Tong et al., 2021). The upregulation of efflux genes also contributes to the persistence of chlorine-treated cells (Hou et al., 2019). In our current study, *qacE*Δ*1* was tightly linked to elevated MIC for NaClO. Previous studies had reported that the *qacE*Δ*1* gene was the most frequently present in *E. coli* (69.70%), followed by *K. pneumoniae* (50.00%) and *Salmonella* (39.62%), respectively. The *qacE*Δ*1* gene is common in enteric bacteria and encodes an efflux pump conferring resistance to chlorine disinfectants via an electrochemical proton gradient (Wu et al., 2015). The gene is also frequently associated with mobile genetic elements such as class I integrons resulting in co-selection of antibiotic resistance genes (Hu et al., 2018).

### Cross-Resistance Between Chlorine Disinfectants and Antibiotics

Cross-resistance between chlorine and antibiotics has become a particular concern due to the possible contribution to the persistence of antibiotic resistance despite antibiotic withdrawn (Liao et al., 2020). In this study, exposure to chlorine resulted in cross-resistance to ceftiofur, tetracycline, ciprofloxacin, and florfenicol. Expression of multidrug efflux pumps is a major mechanism mediating such cross-resistance (Hou et al., 2019; Jin et al., 2020; Puangseree et al., 2021). The over-expression of the drug efflux pump MexEF-OprN in *Pseudomonas aeruginosa* following chlorine exposure promoted resistance to diverse antibiotics including tetracyclines, fluoroquinolones, β-lactams, chloramphenicol, macrolides, novobiocin, trimethoprim, and sulfonamides (Lister et al., 2009). This directly linked increased antibiotic resistance to chlorine exposure. Similarly, pretreatment of *S.* Enteritidis with chlorine, sodium nitrite, sodium benzoate or acetic acid induced the over-expression of the marRAB operon, a global antibiotic resistance regulator involved in the production of AcrAB efflux pumps that extrude antibiotics (Potenski et al., 2003). The development of VBNC bacteria via chlorination increases their resistance to antibiotics (Lin et al., 2017). Chlorine-tolerant injured bacteria that are physiologically competent cells present higher plasmid transformation frequencies than the corresponding untreated bacteria. Since the transferable plasmids released from killed sensitive antibiotic resistant bacteria have a consistent resistance to degradation through disinfection, the chlorination process can promote the horizontal transfer of the released plasmid into chlorine-injured bacteria through natural transformation. This leads to the enrichment of antibiotic resistance genes in viable bacteria (Jin et al., 2020). Therefore, using chlorine disinfectant could provide selection pressure for strains that acquire antibiotic resistance.

## Conclusion

*Salmonella* spp. from poultry supply chains and possessed tolerance to NaClO at MIC > 256 mg/L in > 90% of the 172 isolates. These tolerant strains displayed higher *D* values and a more integrated cellular shape. The presence of NaClO tolerance genes and the MIC for NaClO were positively correlated especially for *qacEΔ1* (*r* = 0.66, *p* < 0.0001). Significant positive relationships existed between NaClO and the antibiotics ceftiofur, tetracycline, ciprofloxacin, and florfenicol indicating that chlorine-tolerant bacteria were more likely to also be antibiotic resistant. The generated data could provide parts of the input data for microbial risk assessment of *Salmonella* with different chlorine tolerance profile to capture the variability in the strains of interest while decreasing the uncertainty in some model input parameters.

## Data Availability Statement

The original contributions presented in the study are included in the article/supplementary material, further inquiries can be directed to the corresponding author/s.

## Author Contributions

WW and HY: writing—review and editing. XX and LB: investigation and writing—original draft preparation. SW and LL: data curation. XQ and JZ: software. YX, BT, and YL: resources. All authors contributed to manuscript revision, read, and approved the submitted version.

## Conflict of Interest

The authors declare that the research was conducted in the absence of any commercial or financial relationships that could be construed as a potential conflict of interest.

## Publisher’s Note

All claims expressed in this article are solely those of the authors and do not necessarily represent those of their affiliated organizations, or those of the publisher, the editors and the reviewers. Any product that may be evaluated in this article, or claim that may be made by its manufacturer, is not guaranteed or endorsed by the publisher.

## Footnotes

1
https://cge.cbs.dtu.dk/services/ResFinder/gene_overview.php
